# Wheat Straw Lignin Nanoparticles as Active Filler in Thermoplastic Starch Films

**DOI:** 10.3390/polym17172308

**Published:** 2025-08-26

**Authors:** Florian Zikeli, Franco Dominici, Marco Rallini, Sebastian Serna-Loaiza, Walter Wukovits, Anton Friedl, Michael Harasek, Luigi Torre, Debora Puglia

**Affiliations:** 1 Civil and Environmental Engineering Department, UdR INSTM, University of Perugia, Strada di Pentima 4, 05100 Terni, Italy; franco.dominici@unipg.it (F.D.); marco.rallini@unipg.it (M.R.); luigi.torre@unipg.it (L.T.); debora.puglia@unipg.it (D.P.); 2 Research Unit of Thermal Process Engineering and Simulation, Institute of Chemical, Environmental and Bioscience Engineering, TU Wien, Getreidemarkt 9/166, 1060 Vienna, Austria; sebastian.serna@tuwien.ac.at (S.S.-L.); walter.wukovits@tuwien.ac.at (W.W.); anton.friedl@tuwien.ac.at (A.F.); michael.harasek@tuwien.ac.at (M.H.); 3 Christian Doppler Laboratory for Next-Generation Wood-Based Biocomposite, Institute of Chemical, Environmental and Bioscience Engineering, TU Wien, Getreidemarkt 9/166, 1060 Vienna, Austria

**Keywords:** starch, lignin, antioxidant activity, packaging films, film extrusion

## Abstract

Starch and lignin are promising biopolymers for the production of biodegradable biocomposite materials. The possibility of processing starch into thermoplastic materials qualifies it as a starting material for the preparation of thermoplastic packaging films, and the combination with lignin can even out some inherent weak points of starch, such as moisture and water sensitivity, and can add additional features like antioxidant activity. Lignins from herbaceous biomass carry building blocks that are not found in wood lignins and are known for their bioactivity, such as *p*-coumaric acid or ferulic acid. In this work, a protocol was developed to initially prepare hybrids of wheat starch granules and lignin nanoparticles, which were then plasticized using glycerol in an extrusion process to produce thin films. The lignin-containing thermoplastic starch films showed higher Young’s moduli and less elongation at break compared to neat thermoplastic starch films, while tensile strength remained at the level of the neat films. Thermal stability was slightly increased by lignin addition, and oxygen transmission rates were low for lignin contents as low as 1 wt%. The hydrophobicity of the lignin-containing films increased strongly, and they showed an elevated antioxidant activity over several hours, which was also maintained after 24 h. The preparation of hybrid wheat starch lignin particles was successfully tested for the extrusion of thermoplastic starch films with improved thermomechanical properties, decreased water sensitivity, and prolonged antioxidant activity.

## 1. Introduction

Starch is an attractive matrix for non-food industrial applications, due to its high abundance, low cost, its inherent biodegradability, and the fact that it can be processed with equipment that is conventionally used in the plastics industry [1]. More than half of the isolated starch is dedicated to non-food applications, such as glues, flocculants, packaging materials, plastics, organic chemicals, or pharmaceuticals [2,3]. In the case of wheat starch, two different types of starch granules are present, which differ in their chemical composition and hence also their properties. While A-starch contains larger granules, B-starch consists of smaller granules with a round shape; contains more proteins, lipids, and sugars; and is therefore of lower quality and problematic for food production. Thus, wheat B-starch presents a valuable starting material for the production of starch-based bioplastics, which is not in direct competition with starch for food production [3]. Starch is a high-molar-mass carbohydrate polymer that consists of a linear fraction called amylose, which consists of α-1,4-linked glucose units, and a branched fraction named amylopectin, which is built up by shorter α-1,4-linked glucose chains that are interconnected by α-1,6 glycosidic bonds [1]. The proportion of amylose and amylopectin depends on the botanical origin of the starch and influences its physical properties. Thus, starches can be classified based on their amylose content into waxy starches (amylose content < 15%), regular (amylose content 20–35%), and high-amylose (amylose content > 40%) starches [4].

The possibility of producing thermoplastic starch films has been known for about 30 years, but the utilization of the developed materials for food packaging has faced obstacles, such as swelling and partial dissolution in humid conditions [5]. The phenomenon that renders starch thermoplastic is its gelatinization at a certain temperature: In the presence of water or other plasticizers, the crystalline structure in the starch granules is irreversibly destroyed by the cleavage of hydrogen bonds and the eventual attachment of water molecules to the hydroxyl groups in the starch molecule. This infiltration of water into the starch macrostructure, in consequence, leads to increased swelling of the starch and dissolution of the starch crystalline regions. Otherwise, when using extrusion, starch can also be plasticized in the absence of water due to high shear strength and high pressure, and in the presence of a plasticizer like glycerol, which additionally reduces starch degradation under extrusion processing conditions [1].

Lignin is an interesting candidate as an additive to biopolymer composites to confer antioxidant, UV-shielding, and antimicrobial properties in packaging films made from biopolymers, such as thermoplastic starch (TPS), especially when applied in nano-size [2,6]. As the second most abundant biopolymer after cellulose, lignin is produced as a byproduct in biorefineries or the pulp and paper industry in vast amounts. Lignin chemically consists of up to three different monomeric units called monolignols, which are the differently substituted phenylpropanoid units called *p*-coumaryl, coniferyl, and sinapyl alcohol, that combine via radical polymerization to a crosslinked macromolecule built up from guaiacyl (G) units (softwoods), G and syringyl (S) units (hardwoods), and G, S, and *p*-hydroxyphenyl (H) units (non-wood plants) [7,8,9]. Besides their natural origin, the lignin extraction method also influences lignin structure, properties, and availability. Thus, lignin isolated via the Kraft process, which is the most used pulping process, is available in high abundance but contains sulfur and is insoluble in water. In contrast, lignosulfonates deriving from the sulfite process are water-soluble, but can contain up to 8 wt% of sulfur. Instead, the Organosolv process and the soda process provide sulfur-free lignins [10]. In contrast to lignin deriving from wood, lignin from herbaceous biomass such as wheat straw is characterized by additional structural features, like *p*-hydroxycinnamic acids (*p*HCAs), which can be present either incorporated into the lignin macromolecule or as ester-bound pendant groups on lignin side chains [4,11]. These structural features could promote interactions between the active filler and the biopolymer matrix during film formation, providing improved thermal and mechanical properties. Further, they could enhance the antioxidant potential of the final product, which is of great interest for the food packaging industry.

In general, lignin was added to starch products in order to compensate for characteristic weak points of starch, such as moisture and water sensitivity, thermolability, UV sensitivity, and mechanical strength, and to add new properties like antioxidant activity [2]. Early works included Kraft lignin into wheat starch plasticized with water and concluded that lignin could only be added until a content of 20 wt%; otherwise, mechanical properties like resistance to elongation would decrease [5]. The mechanical properties and the water absorption of TPS can be improved by adding lignin, as demonstrated by Kaewtatip and Thongmee [12], who prepared lignin-reinforced TPS composites using 5% Kraft lignin and esterified lignin by compression molding. Concordant results were reported by Bhat et al. [13], who prepared starch films from sago palm starch, glycerol, and lignin isolated from black liquor from oil palm. Espinoza Acosta et al. [14] prepared starch–lignin composite films by solvent casting, blending Organosolv wheat straw lignin with wheat starch matrix. In contrast to other studies, lignin addition resulted in a significant decrease in tensile strength and elastic modulus but increased elongation at break. Additionally, the starch films acquired antioxidant activity via the added lignin. These authors also observed an increased thermal stability at higher temperatures with increased lignin contents, although degradation onset temperatures were lower when lignin was added. Wang et al. [15] prepared thermoplastic cassava starch reinforced with lignin via melt-blending using an extruder, followed by hot-pressing, and registered a strong increase in tensile strength and a reduction in the elongation at break. Similarly, lignin addition increased tensile and impact strength of a starch/poly (butylene adipate-co-terephthalate) blend produced by melt compounding, while it also conferred antibacterial properties to the final product aimed for the production of packaging materials [16].

**Figure 1:**
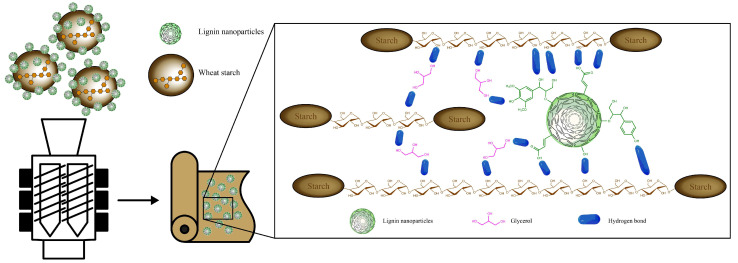
Illustrative scheme of the preparation of hybrid particles of wheat straw granules in micrometer scale and wheat straw lignin nanoparticles used for the hot melt extrusion of thermoplastic starch packaging films. Interactions between starch, lignin, and glycerol via hydrogen bonding are illustrated in the insert.

Starch–lignin interactions are generally developed via hydrogen bonding between hydroxyl groups on the starch amylose backbone or amylopectin branches on the one side and aliphatic, carboxylic, and phenolic hydroxyl groups, as well as carbonyl groups of lignin on the other side, as pointed out in the reference literature [2,17,18,19,20]. The interactions between starch, lignin, and glycerol, which was applied as a plasticizer, are schematically illustrated in Figure 1.

Lignin was also incorporated into starch films on a nanoscale, preparing composite films using solution casting [17]. Lignin addition improved the hydrophobicity of the films, increased thermal stability, and conferred UV-blocking properties to the produced lignin–starch films. Similarly, Sun et al. [18] prepared potato starch films with lignin nanoparticles (LNPs) using glycerol as a plasticizer and a solution casting method. The prepared packaging films showed increased tensile strength and elastic modulus in the range of 1–5% of added LNPs, with a maximum effect when 2–3% of LNPs were added. Other improvements were the acquired antioxidant activity and UV irradiation shielding. LNPs from residual wheat starch, which was used for bioethanol production, were applied as reinforcing agents in starch films by Roostazadeh et al. [21], and they registered a tensile strength and elastic modulus increase when adding 20% LNPs.

Through the formation of nanoparticles, lignin’s inherent properties, like antioxidant activity, can be improved due to their higher surface-to-area ratio compared to the starting lignin molecule [22]. Spherical lignin particles spread better on surfaces and can be more evenly dispersed in media than crude lignins, and are therefore expected to have a higher compatibility with the polymer matrix, due to their well-defined surface chemistry, morphology, and narrow particle size distribution [23].

Although lignin has been widely applied in TPS materials, as pointed out above, the literature reports regarding LNPs incorporated into TPWS films are rather scarce. To the best of our knowledge, the approach presented in this work, using nanoprecipitation of lignin on starch granules in dispersion and the eventual use of the so-produced hybrid wheat starch lignin nanoparticles (hWSLP) for the production of TPS-extruded films, is a novelty. The intention for the development of this novel protocol for the formation of LNPs was to facilitate their application inside a biopolymer matrix and improve the interactions between the matrix and the filler by their formation directly on the matrix particles. The hWSLPs were characterized by FESEM, and thermomechanical behavior and radical scavenging activity of the produced extrusion films were also evaluated.

## 2. Materials and Methods

### 2.1. Materials

Commercial wheat straw lignin from alkaline pulping (Protobind1000) was supplied from PLT Innovations AG, Rüschlikon, Switzerland, and was named SL hereafter. Wheat starch was purchased from Caesar& Loretz GmbH (Hilden, Germany). Glycerol, EtOH, MeOH, gallic acid, 2,2-diphenyl-1-picrylhydrazyl (DPPH), and sodium carbonate were purchased from Merck KGaA (Darmstadt, Germany).

### 2.2. Preparation of Organosolv Wheat Straw Lignin

Organosolv wheat straw lignin (OL) was prepared using ethanol–water for Organosolv extraction at 180 °C for 60 min, following [24] and separated by precipitation after evaporation of ethanol, followed by repeated washing with acidified water (pH 2), centrifugation, and freeze-drying.

### 2.3. Preparation of Hybrid Wheat Starch–Lignin Nanoparticles

Hybrid wheat starch–lignin nanoparticles (hWSLPs) were prepared via the drop-wise addition of 10 mL of a lignin solution (20 g/L and 60 g/L) in aqueous acetone (60 vol%) into 150 mL of an aqueous dispersion of wheat starch (92.4 g/L and 90.5 g/L) during vigorous magnetic stirring. Stirring was continued for 1 h, and the resulting dispersions of lignin nanoparticles and wheat starch granules were freeze-dried. The proportion of lignin to wheat starch was 1.44 wt% of lignin for the hWSLPs eventually used for the preparation of the 1 wt% lignin-containing wheat starch films, and 4.42 wt% of lignin for the hWSLPs used for the 3 wt% lignin-containing wheat starch films. In a modified protocol, the resulting lignin–wheat starch dispersions were heated to 70 °C to initiate wheat starch gelatinization, and the temperature was kept at 70 °C for 30 min while stirring until the viscosity increased, and when cooling down, a gel was formed. The resulting wheat starch gels containing LNPs were freeze-dried.

### 2.4. Hot Melt Extrusion of Wheat Starch–Lignin Films

Wheat starch–lignin films were realized by using a twin-screw micro-extruder (DSM Xplore 5&15 CC Micro Compounder/Film Device, Xplore Instruments BV, Sittard, The Netherlands). During the mixing/plasticization step, the screw speed was set at 60 rpm, with a temperature profile of 135–140–145 °C for 180 s. Eventually, film extrusion was conducted while maintaining a head force of 2000 N or at least the equivalent of 18 rpm. Extrusion films were cooled in an air stream at the exit of the extruder and collected on rolls with constant speed and constant torque, resulting in films with a thickness of 230–700 µm. Masterbatches of 30 g were prepared from the hWLSPs preparations containing 30 wt% glycerol as plasticizer and lignin in concentrations of 1 wt% and 3 wt%, respectively (Figure 1). The films prepared from OL containing hWSLPs were labeled as OL1 and OL3, and OL1gel and OL3gel for the respective pre-gelatinized hWSLPs. Likewise, films from SL were labeled as SL1, SL3, SL1gel, and SL3gel.

### 2.5. Analytical Methods

The formulations containing wheat starch and LNPs were analyzed by field emission scanning electron microscopy (FESEM, Supra 25, Carl Zeiss AG, Feldbach, Switzerland) at an operating voltage of 2.5 kV. A drop of the dispersions containing hWSLPs from WSL and SL was air-dried on silica wafers and gold-sputtered before analysis. TPWS extrusion films were analyzed by FESEM regarding their fracture area after breaking film samples in liquid nitrogen.

The utilized lignin samples and the prepared TPWS/lignin films were characterized using thermogravimetric analysis (TGA) using an Exstar 6300 TGA instrument (Seiko Instruments Inc., Chiba, Japan). Approximately 5–10 mg samples were heated from 30 °C to 900 °C at a heating rate of 10 °C/min under a nitrogen atmosphere. The temperature of maximum degradation rate (DTG_max_) was obtained from derivative thermogravimetric data (DTG).

Mechanical properties of the TPWS-extruded films were evaluated via tensile strength tests using an LR30K universal testing machine (Lloyd Instruments/Ametek Inc., Berwyn, IL, USA) following UNI ISO 527. Rectangular film samples (100 mm × 10 mm), after conditioning at 53% relative humidity, were tested using a load cell of 500 N, an initial gauge length of 50 mm, and a crosshead speed of 5 mm/min. Based on the stress–strain curves, the average tensile strength, Young’s modulus, and elongation at break were calculated.

The total phenolic content (TPC) of the TPWS extrusion films was determined using the aqueous EtOH extracts following the Folin–Ciocalteu protocol and using gallic acid as a reference standard. The diluted (1:1) extracts (0.2 mL) were mixed with 1.8 mL of distilled water and 0.2 mL of Folin–Ciocalteu reagent. After 6 min, 2 mL of 7% sodium carbonate solution was added, and the sample was incubated in the dark for 90 min before reading the absorbance at 750 nm using a Lambda 35 UV-VIS spectrometer (PerkinElmer Inc., Waltham, MA, USA). Quantification of TPC was performed relative to a gallic acid standard curve, and TPC was expressed as mg GAE per g of TPWS-extruded film. TPC was also determined for the utilized lignin samples using DMSO solutions in a final concentration of 1 mg/mL.

The oxygen transmission rate (OTR) was determined using a Systech 8000 oxygen permeation analyzer (Systech Instruments Metrotec S.A., Lezo, Spain). Introducing pure oxygen (99.9%) into the upper half of the measurement chamber and nitrogen into the lower half with an oxygen sensor, the oxygen volumetric flow rate per unit area was continuously monitored until reaching a steady state. OTR values were normalized for 1 mm thick films in order to compare the OTR values determined for the different prepared TPWS films containing lignin.

Contact angle measurements were conducted using an FTA1000 Analyzer System and Drop Shape Analysis SW21 and FTA32 2.0 software (First Ten Angstroms Inc., Newark, NJ, USA) following the method reported in [25]. At least five repetitions were conducted for each film formulation, dripping one drop of water on the film surface using a syringe, followed by immediately taking a photograph with the built-in camera.

Radical scavenging activity (RSA) of the lignin-containing TPWS films was determined following the method applied by [26] and was expressed as residual DPPH. A sample (1.0 g) of the TPWS films was cut into small pieces and covered with 2 mL of EtOH (60 vol%) for an extraction of 24 h. Extraction was repeated once to ensure full extraction of phenolic compounds contained in the TPWS extrusion films. The resulting supernatant was used for a radical scavenging activity assay using DPPH. A sample of 50 µL of the EtOH extracts was mixed with 3 mL of DPPH solution in MeOH (24 mg/L) and stored in the dark at room temperature for 60 min. Absorbance at 517 nm was measured using a Lambda 35 UV-Vis spectrometer (PerkinElmer Inc., Waltham, MA, USA). RSA was calculated relative to a gallic acid standard curve, and was expressed as mg gallic acid equivalents (GAE) per g of TPWS extrusion film. The RSA of TPWS film samples was determined following the method described by [27]. Pieces of the TPWS films in a size of 1 cm^2^ were directly immersed in 3 mL of the DPPH solution, and the absorbance was measured after 30, 60, 90, 120, 180, and 240 min, in order to calculate the RSA and the residual DPPH° following Equation (1):

(1) $$RSA\;\left(\%\right)=\frac{A_{control}-A_{sample}}{A_{control}}\times 100=1-Residual\;DPPH{}^{\circ}$$

## 3. Results

### 3.1. Characterization of Hybrid Wheat Starch Lignin Particles

**Figure 2:**
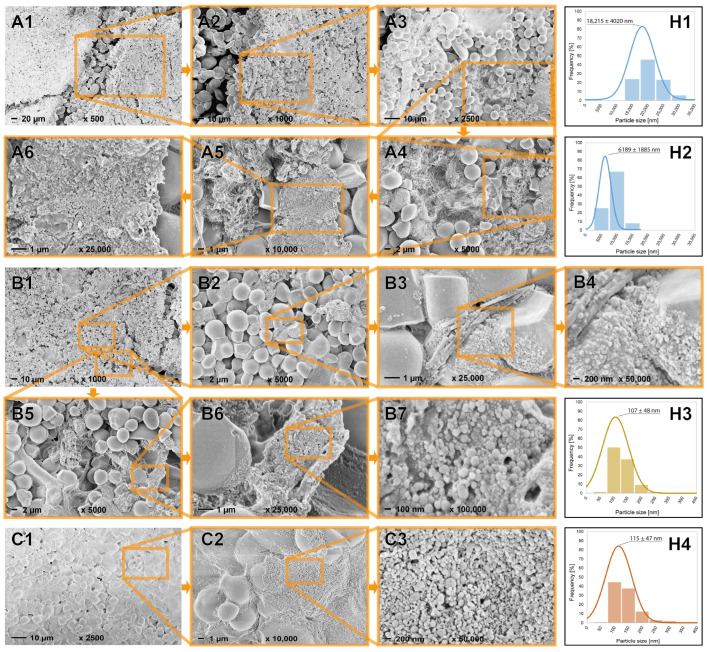
FESEM images of freeze-dried hybrid wheat starch–lignin particles in different magnifications and respective histograms of particle size distributions of large wheat starch granules (**A1**,**A2**,**H1**), small wheat starch granules (**A3**–**A6**,**H2**), OL nanoparticles (**B1**–**B7**,**H3**), and SL nanoparticles (**C1**–**C3**,**H4**).

**Figure 3:**
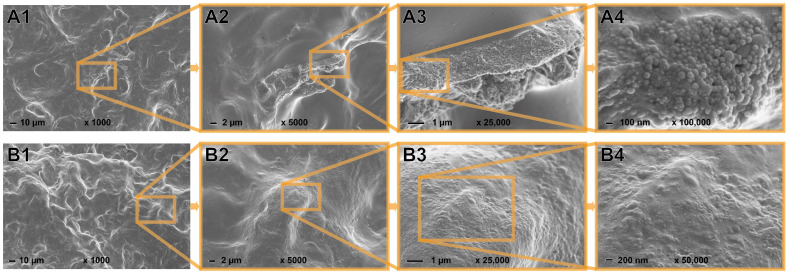
FESEM images of freeze-dried pre-gelatinized hybrid wheat starch lignin particles in different magnifications from OL (OL3gel, **A1**–**A4**) and SL (SL3gel, **B1**–**B4**).

The prepared hWSLPs were characterized by FESEM, and the acquired images, plus their respective particle size distributions, are presented in Figure 2. Wheat starch granules are present in two distinct particle size fractions, one having a maximum of around 15–20 µm (Figure 2(H1)) and the second one showing a maximum of around 5–10 µm (Figure 2(H2)). The calculated average particle diameters are 18,215 ± 4020 µm for the bigger fraction of wheat starch granules and 6189 ± 1885 µm for the smaller fraction of wheat starch granules, respectively. Surrounding the wheat starch granules, lignin particles in the nanoscale are visible, apparently holding together the wheat starch granules of the lower particle size fraction (Figure 2(A4–A6)). FESEM analysis showed the presence of polysaccharide fibers in the OL sample used in this study, which becomes more evident at higher magnifications (Figure 2(B2–B5)). In contrast, no fiber residues were identified in the FESEM images of the particles prepared using SL, which was used as a commercial lignin sample (Figure 2(C1–C3)). Regarding their particle sizes, the nanoparticles prepared from the two different lignin samples showed almost identical maxima of around 110 nm (Figure 2(H3,H4)). The antisolvent method used in this study, therefore, successfully produced nanoparticles with a very narrow particle size distribution, apparently independently of the lignin type, and in a high yield since all of the starting material is transformed into hybrid particles and no separation steps are involved (Table 1). The pre-gelatinization protocol resulted in a completely different morphology of the hWSLPs, as shown in Figure 3. A continuous starch phase is dominating in both OL3gel and SL3gel, and only where the phase is broken up, underlying LNPs can be identified in the case of OL3gel (Figure 3(A3,A4)). In the case of SL3gel instead, no spots with an open starch phase were identified in the zone investigated by SEM, but on the surface, there were still signs of underlying LNPs (Figure 3(B3,B4)).

**Figure 4:**
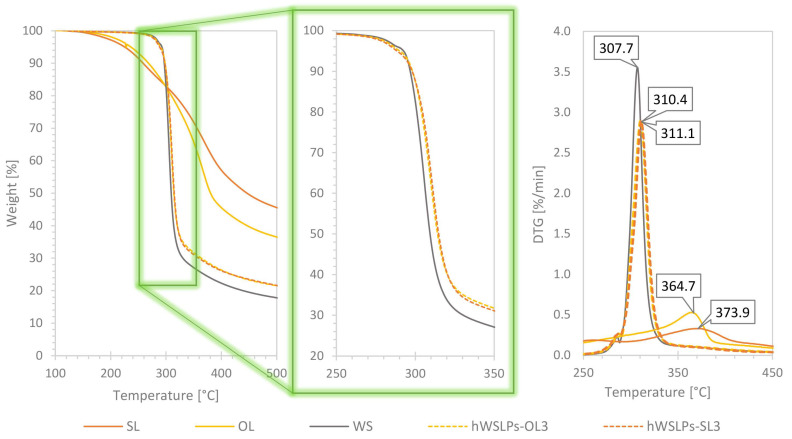
Thermogravimetric and differential thermogravimetric (DTG) curves of wheat starch (WS), Organosolv lignin (OL, soda lignin (SL), and two samples of hybrid wheat starch lignin nanoparticles (hWSLPs-OL3, hWSLPs-SL3) from OL and SL at 3 wt% content, respectively.

The starting materials WS, OL, and SL, as well as the prepared hWSLPs, were characterized by TGA. Starch and lignin show different thermal behavior, with lignin having a lower onset of thermal degradation (T_onset_ = 258.5 °C for SL, and T_onset_ = 268.3 °C for OL) compared to WS (T_onset_ = 296.8 °C) but a considerably higher residue at 500 °C (Figure 4). The same observations were reported by [17], concluding a better thermal stability of lignin for temperatures > 300 °C. The higher thermal stability of lignin is due to the fact that the cleavage of ether-type inter-unit and C-C bonds of lignin occurs at higher temperatures than the cleavage of the glycosidic bonds in starch [28]. The addition of lignin to the hWSLPs, in consequence, resulted in a lower T_onset_ compared to pure WS and a higher residue at 500 °C (Table 1). Regarding the maxima of the DTG curves, a lower temperature was determined for WS (307.7 °C) compared to the two lignin samples, OL (364.7 °C) and SL (373.9 °C). In addition, the maximum of the DTG curve of WS is much sharper than that of the lignin samples, which most probably is caused by the more homogenous structure of WS compared to lignin, causing the thermal degradation of WS to happen in a narrower temperature window [29]. With the addition of 1 and 3 wt% lignin in the prepared hWSLPs, the DTG_max_ increased compared to pure WS, indicating an improvement in the thermal behavior of WS due to the added lignin. Interestingly, the difference in the DTG_max_ temperatures of the two starting lignins was not encountered in the respective hWSLPs, which showed their maxima around 310–311 °C, independent of lignin type and lignin content. Similarly, the T_onset_ of the different hWSLPs was in a narrow range of 297.3–299.5 °C, indicating that lignin type and lignin content in the range of 1–3% were not decisive regarding thermal behavior when the lignin was applied in the form of LNPs.

### 3.2. Thermal and Mechanical Characterization of Thermoplastic Wheat Starch–Lignin-Extruded Films

**Figure 5:**
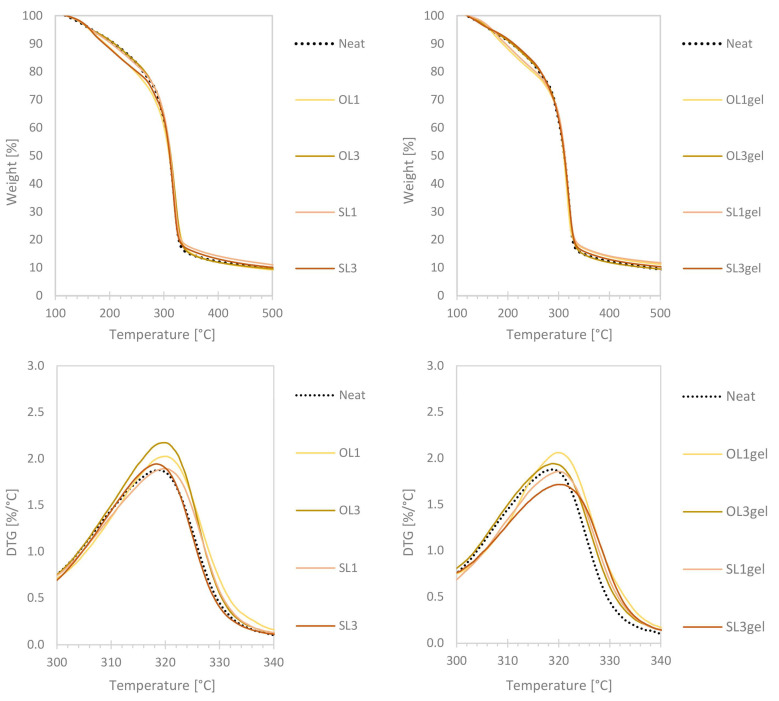
(**above**): TG curves of the thermoplastic wheat starch (TPWS) films without lignin (Neat) and with 1 wt% and 3 wt% of Organosolv lignin (OL1, OL3) and soda lignin (SL1, SL3), as well as in pre-gelatinized form (OL1gel, OL3gel, SL1gel, and SL3gel). (**below**): Differential thermograms of the different prepared TPWS films.

The TPWS films with the two different lignins at different concentrations, with and without pre-gelatinization, were characterized by TGA. The addition of lignin led to a lower onset and a slight increase in residual weight at 500 °C (Figure 5 above). As a second observed effect, the DTG_max_ was shifted slightly to higher temperatures when lignin was added (Figure 5 below). The effect of lignin addition on the thermal behavior of the TPWS films was not as pronounced as that of the pure starch film sample (Neat), indicating that the incorporation of LNPs into the TPWS film matrix did not create major disturbances, which would result in significantly different thermal behavior. In contrast, the changed thermal behavior was more evident in the case of the hWSLPs (Figure 4). The lignin effect was possibly compensated in the TPWS films by the presence of glycerol in the TPWS matrix, acting as a plasticizer for the starch. Comparing the TG curves of WS (Figure 4) and neat TPWS film (Figure 5), the lower thermal stability of the glycerol-containing films at temperatures below 300 °C is evident. The similar thermal behavior of the lignin-containing TPWS films produced in this study is in contrast to other studies where higher lignin contents (5%) were utilized, and the thermal behavior of the lignin-containing films there derived much more from the control starch films. Espinoza Acosta et al. [14] explained the strongly reduced DTG_max_ with discontinuity of the starch matrix caused by the addition of lignin. It could therefore be assumed that the protocol used here, where hWSLPs were produced prior to adding glycerol and film extrusion, could have helped to form a continuous matrix of the produced TPWS films, leading to a negligible effect on the DTG_max_ of the lignin-containing films. In another recent study, instead, the DTG_ma_x values significantly increased with growing contents of LNPs from 1 to 3 wt% [30], an effect that was observed to a lesser extent also in the study presented herein. The thermomechanical behavior is almost identical for the TPWS films prepared from the pre-gelatinized hWSLPs, with a slight increase in the maximum degradation temperature DTG_max_ for the films that contain lignin. One significant difference is the height of the DTG_max_, which is lower in the case of the films made from the pre-gelatinized hWLSPs, indicating that the pre-gelatinized hWSLPs cause a slower thermal degradation of the resulting extruded films. There was a trend observed regarding the onset temperatures T_onset_ of the TPWS films with different lignin contents and when prepared from pre-gelatinized hWSLPs (Table 2). For OL1 and OL1gel, T_onset_ was the same as for the neat film, while it decreased for OL3. For OL3gel instead, T_onse_t increased by 17.2 °C compared to the neat film, apparently due to the use of already pre-gelatinized starch for film extrusion. The same trend was observed for SL, where To_nset_ was highest for the film SL3gel. For SL1 and SL1gel, T_onset_ was 10 °C higher than for the neat film, which could be attributed to the lignin-type SL that is characterized by a high thermostability and a DTG_max_ of 373.9 °C (Table 1), while for SL3, T_onset_ was lowest, as it was also observed for OL.

The addition of lignin affected the oxygen permeation behavior of the TPWS films depending on the lignin concentration and the preparation method (Table 2). An increased lignin content of 3 wt% caused a rather strong increase in the OTR values, both for OL and for SL, while the films with 1 wt% lignin maintained low OTR values in the range of the requisites for oxygen barrier packaging material, which is reported as lower than 10 cm^3^m^−2^day^−1^atm by [31]. In particular, the TPWS film OL1 showed a lower OTR value than the reference neat TPWS film. Pre-gelatinized hWSLPs apparently had no concentration-dependent effect on the determined OTRs, but the lignin type apparently influenced the oxygen barrier properties, with SL giving higher values than OL. The increase in OTR with the addition of lignin is in contradiction to studies that observed the opposite effect when adding 4 wt% of lignin microparticles to thermoplastic starch and pectin films [32]. There, the OTR measured for the lignin-containing films was lower than that of the neat thermoplastic starch and pectin films.

**Figure 6:**
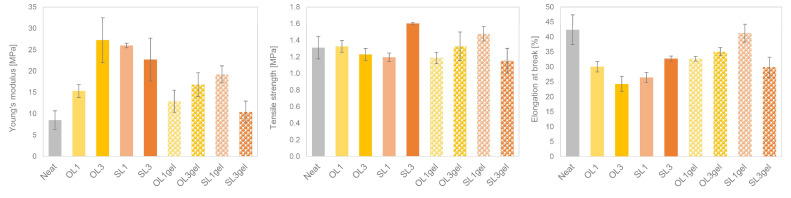
Young’s modulus, tensile strength, and elongation at break of pure TPWS extrusion film (Neat), and TPWS extrusion films containing wheat straw Organosolv lignin (OL) and soda lignin (SL), respectively, in different concentrations.

With the addition of lignin, there was a strong increase in Young’s modulus. This increase was most evident for OL3, SL1, and SL3 (Figure 6). Interestingly, in the case of SL, the increase was more pronounced for the TPWS film samples with only 1% lignin added. For the TPWS film samples where pre-gelatinized WS was applied, the increase in Young’s modulus was less pronounced, and the sample SL3gel, again, showed a lower Young’s modulus than the SL1gel film. Tensile strength remained in the range of neat TPWS films for all lignin-containing films, and was significantly higher for two samples: SL3 and SL1gel. Elongation at break was strongly reduced with the addition of lignin by around 10%. The TPWS films with pre-gelatinized WS tended to have a lower reduction in elongation at break, but still showed lower values than the neat TPWS film, with the exception of the sample SL1gel, where elongation at break stayed in the range of the neat TPWS film. This is in contrast to reports by [30], where a significant increase in the elongation at break was observed with increasing LNP contents. It has to be noted that the neat starch film produced by [30] showed quite different mechanical properties than the neat starch film produced here. While there, the elongation at break was as low as 12.1% and the tensile strength was 3.7 MPa; here, the elongation at break was around 40% and the tensile strength was as low as 1.3 MPa. The different film-forming methods (casting method vs. film extrusion) supposedly produce starch films with diverging mechanical properties, which is also indicated by the film thickness of the respective starch films: neat starch films produced here had an average thickness of around 600 µm, while [30] produced films with around 115 µm thickness.

**Figure 7:**
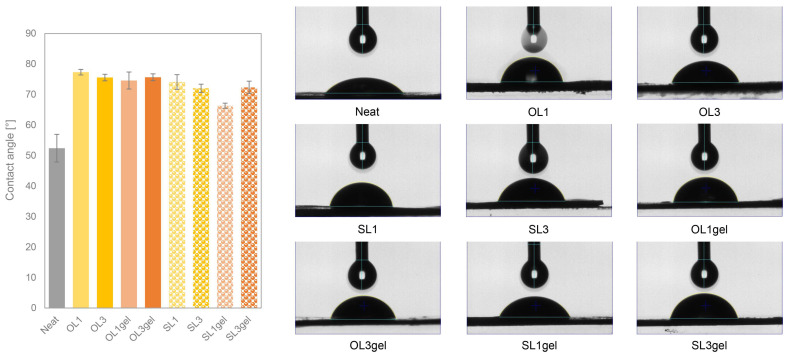
Contact angles of the TPWS films with 1 and 3 wt% lignin content (OL1, OL3, SL1, SL3) and from pre-gelatinized hWSLPs (OL1gel, OL3gel, SL1gel, SL3gel).

Contact angle (CA) measurements of the TPWS films containing lignin showed improved hydrophobicity for both lignins, OL and SL, and the TPWS films, which were prepared from pre-gelatinized hWSLPs. While for the neat TPWS film, the CA was as low as 50°, the CA for lignin-containing TPWS films increased by around 50% (Figure 7). The films prepared with OL showed a higher CA increase than the ones made containing SL, and this effect was more pronounced for the films made from pre-gelatinized hWSLPs. These results are in concordance with the CAs reported [10], who reported CAs around 80° for starch films containing 1 and 3% LNPs.

**Figure 8:**
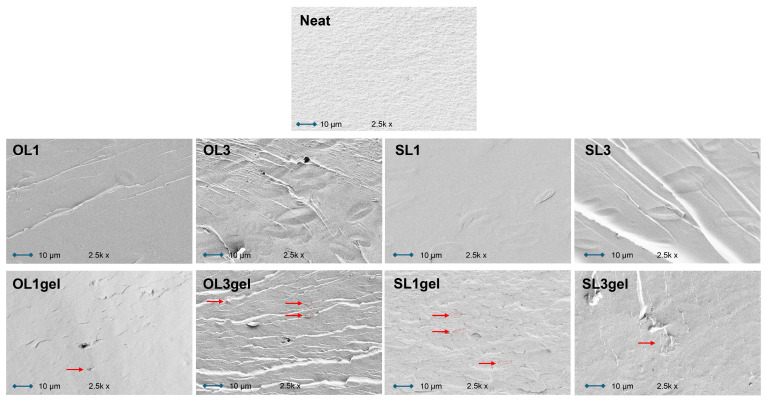
SEM images of the cross-sections of broken TPWS films with 1 and 3 wt% of Organosolv (OL1, OL3) and Soda lignin (SL1, SL3), and of the respective TPWS films prepared from pre-gelatinized hybrid wheat starch lignin particles (OL1gel, OL3gel, SL1gel, and SL3gel). Red arrows and circles indicate lignin nanoparticle agglomerations.

The structure of the neat TPWS cross-section was dense and smooth (Figure 8). The clean film’s surface micrograph showed some granular particles that might have been caused by retrograded or non-plasticized starch molecules. The cross-section of the lignin-containing starch films OL1 and SL1 displays a relatively smooth surface free of pores and cracks, due to the presence of glycerol, which smooths out the surface and confirms the good dispersion of LNPs in the starch matrix. In contrast, cluster aggregation of LNPs and partial phase isolation may be the origin of the cavities and agglomerates seen in OL1gel and SL1gel sample surfaces, which are more noticeable in OL3gel and SL3gel (Figure 8, red arrows and circles). Through shear and interfacial stress concentration zones, and potential detachments between the matrix and additive interface, the different wetting characteristics of the polymer matrix and additive can alter the mechanical properties of composites.

**Figure 9:**
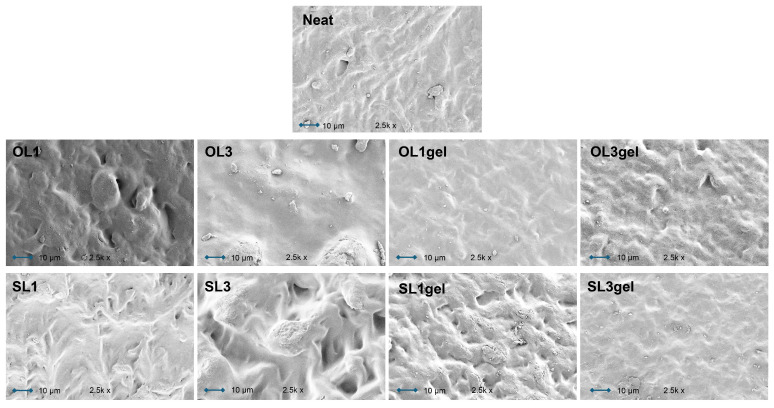
SEM images of the surface of TPWS films with 1 and 3 wt% of Organosolv (OL1, OL3) and Soda lignin (SL1, SL3), and of the respective TPWS films prepared from pre-gelatinized hybrid wheat starch lignin particles (OL1gel, OL3gel, SL1gel, and SL3gel).

By examining the surfaces of the samples, a similar aggregation phenomenon was observed: while the neat TPWS sample has a smooth surface, all of the lignin-containing starch films had uneven and rough surfaces, with SL1 and SL3 samples showing more roughness than OL1 and OL3 (Figure 9). These morphological alterations imply that LNPs may break the continuity of the starch matrix, which may effectively reject water molecules and thus increase the hydrophobicity of the lignin-containing TPWS films. In the case of pre-gelatinized hWSLPs, the micro-cluster aggregations produced by LNPs may be the reason for the formation of micro-convex structures on the surface of the starch film (see sample SL1gel).

### 3.3. Total Phenolic Content and Radical Scavenging Properties of Thermoplastic Wheat Starch–Lignin Extrusion Films

Determination of the TPC of the utilized lignin samples revealed that SL contained a higher amount (+17%) of free phenolic hydroxyl groups compared to OL (Table 3). In the film samples, this difference was not encountered, most probably due to the low lignin concentrations applied in the TPWS batches. Interestingly, the lignin content increase from 1% to 3% was not mirrored by the respective TPC and RSA values determined for EtOH–water extracts of the film samples. This could indicate that lignin extraction from the films by the applied solvent was incomplete, or, instead, lignin incorporation into the TPWS matrix was rather complete, inhibiting lignin extraction by EtOH–water. Higher TPC was found for the TPWS films made from pre-gelatinized hWSLPs for both lignins, OL and SL, which could be caused by lignin hydrolysis at the elevated temperature during the pre-gelatinization protocol. In consequence, higher RSA values were determined for the extracts of the respective films (OL3gel, SL3gel). This effect was stronger in the case of SL.

**Figure 10:**
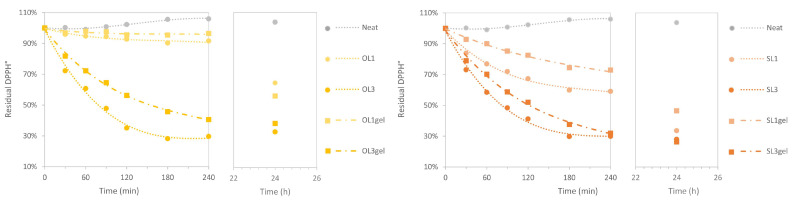
Kinetics of the residual DPPH free radicals during the TPWS film samples immersion experiments in methanolic DPPH° solution.

The RSA of the film samples was directly determined by immersing the film samples in the methanolic DPPH° solution. The residual DPPH° was determined every 30 min for a period of 120 min, and then every 60 min until 240 min experiment time. The respective time-dependent curves of residual DPPH° revealed a slower neutralization of the free DPPH radicals by the TPWS films containing pre-gelatinized hWSLPs (Figure 10). This effect was encountered for both lignins used, OL and SL. The lowest achieved residual DPPH° in the case of OL3 was reached after 180 min, while it was not reached after 240 min in the case of OL3gel. Similarly, for SL3, the minimum of residual DPPH was reached after 180 min, while it was reached after 240 min for SL3gel. For both cases, OL3gel and SL3gel, the residual DPPH° curve was not flat after 240 min experiment time, indicating that the release of the antioxidant agents from the TPWS matrix was not complete at the end of the experiment. In order to confirm this interpretation, the experiments were run for 24 h, and the residual DPPH° was eventually determined. As can be seen from the respective inserts in Figure 10, the residual DPPH for films with the 1 wt% lignin loadings continued to decrease a lot after 24 h, for both OL and SL, while for the 3 wt% loadings it stayed at a level as after 300 min. This observation indicates two situations: first, lignin is released slowly from the TPWS extrusion films produced from hWSLPs, and second, the lignin present in the films with 3% loading can scavenge more DPPH radicals than were present in the used DPPH° methanolic solution. The general shape of the residual DPPH° curves is similar to the ones presented by [27]. However, the antioxidant activity of the TPWS films containing hWSLPs in a lignin content of 3% apparently is higher than the PLA films containing 10% lignin produced by [27]. Considering that the same lignin was used in the case of SL, it could be concluded that the higher antioxidant activity observed in the present study was due to the nanoscale of lignin. Antioxidant activity is one of the properties of lignin, which is enhanced in the nanoscale due to lignin’s smaller particle size and its higher surface-to-weight ratio, which has the effect that more of its antioxidant moieties, such as phenolic hydroxyl groups, are exposed to its chemical surroundings [17,22,23].

## 4. Conclusions

TPWS-extruded films containing LNPs were successfully prepared based on a novel method where hWSLPs consisting of wheat starch granules and LNPs are utilized as the starting material and directly loaded into the extruder after mixing with glycerol as a plasticizer. Additionally, a modified protocol including pre-gelatinization of the wheat starch contained in the hWSLPs was tested, which resulted in extruded films with increased thermal stability based on the determined DTG curves and thermal degradation onset temperatures. While tensile strength remained in the range of neat TPWS films, elongation at break was strongly reduced with the addition of lignin in the form of nanoparticles, and the Young’s moduli of the respective films were increased. CA measurements revealed a strong increase of 50% for the lignin-containing thermoplastic starch films. At low lignin concentration (1%), the TPWS films maintained OTR values that qualify for oxygen barrier packaging material. Additionally, lignin-containing TPWS films acquired strong antioxidant properties, which were characterized by a slow-release effect that maintains radical scavenging activity over several hours.

## Figures and Tables

**Table 1:** Hybrid wheat starch lignin particle (hWSLPs) samples preparation yield, maxima in differential thermogravimetry (DTG_max_), and onset temperatures (T_onset_) of thermal degradation.

Sample		Lignin Content [%]	Isolation Yield [%]	DTG_max_ [°C]	T_onset_ [°C]
WS		-	-	307.7	296.8
SL		-	-	373.9	258.5
OL		-	-	364.7	268.3
hWSLPs	OL1	1	88	310.3	297.7
	OL3	3	90	310.4	298.2
	OL1gel	1	88	310.1	299.3
	OL3gel	3	87	311.6	299.5
	SL1	1	88	309.8	298.1
	SL3	3	90	311.1	297.9
	SL1gel	1	88	309.7	297.3
	SL3gel	3	87	311.1	297.4

**Table 2:** Average thickness of the thermoplastic wheat starch (TPWS) film samples, their maxima derivative thermogravimetry (DTG_max_), and onset temperatures (Tonset) of thermal degradation.

TPWS Film Sample	Lignin Content [wt%]	Thickness [µm]	DTG_max_ [°C]	T_onset_ [°C]	OTR [cm^3^m^−2^day^−1^]
Neat	-	613 ± 90	318.9	190.5	4.6
OL1	1	365 ± 71	320.0	191.7	2.4
OL3	3	315 ± 58	319.6	181.4	70.7
OL1gel	1	327 ± 6	320.0	189.7	34.6
OL3gel	3	338 ± 70	322.0	207.7	21.4
SL1	1	335 ± 7	320.0	203.1	11.2
SL3	3	325 ± 78	318.4	189.7	130.8
SL1gel	1	313 ± 68	319.9	199.5	66.2
SL3gel	3	328 ± 80	320.2	211.9	69.0

**Table 3:** Total phenolic content (TPC) and radical scavenging activity (RSA) of the used Organosolv (OL) and soda lignin (SL), and of the ethanolic extracts from the different TPWS films with different contents of Organosolv (OL) and soda lignin (SL), and from pre-gelatinized hybrid wheat starch lignin particles (gel).

Sample	TPC [mg GAE/g Lignin]	TPC [mg GAE/g Film]	RSA [mg GAE/g Film]
OL	312.4	-	-
SL	366.9	-	-
Neat TPWS	-	0.00	0.00
OL1	-	0.59	0.21
OL3	-	1.11	0.26
SL1	-	0.63	0.20
SL3	-	1.07	0.28
OL1gel	-	0.44	0.19
OL3gel	-	1.50	0.29

## Data Availability

The original contributions presented in this study are included in the article. Further inquiries can be directed to the corresponding author.
